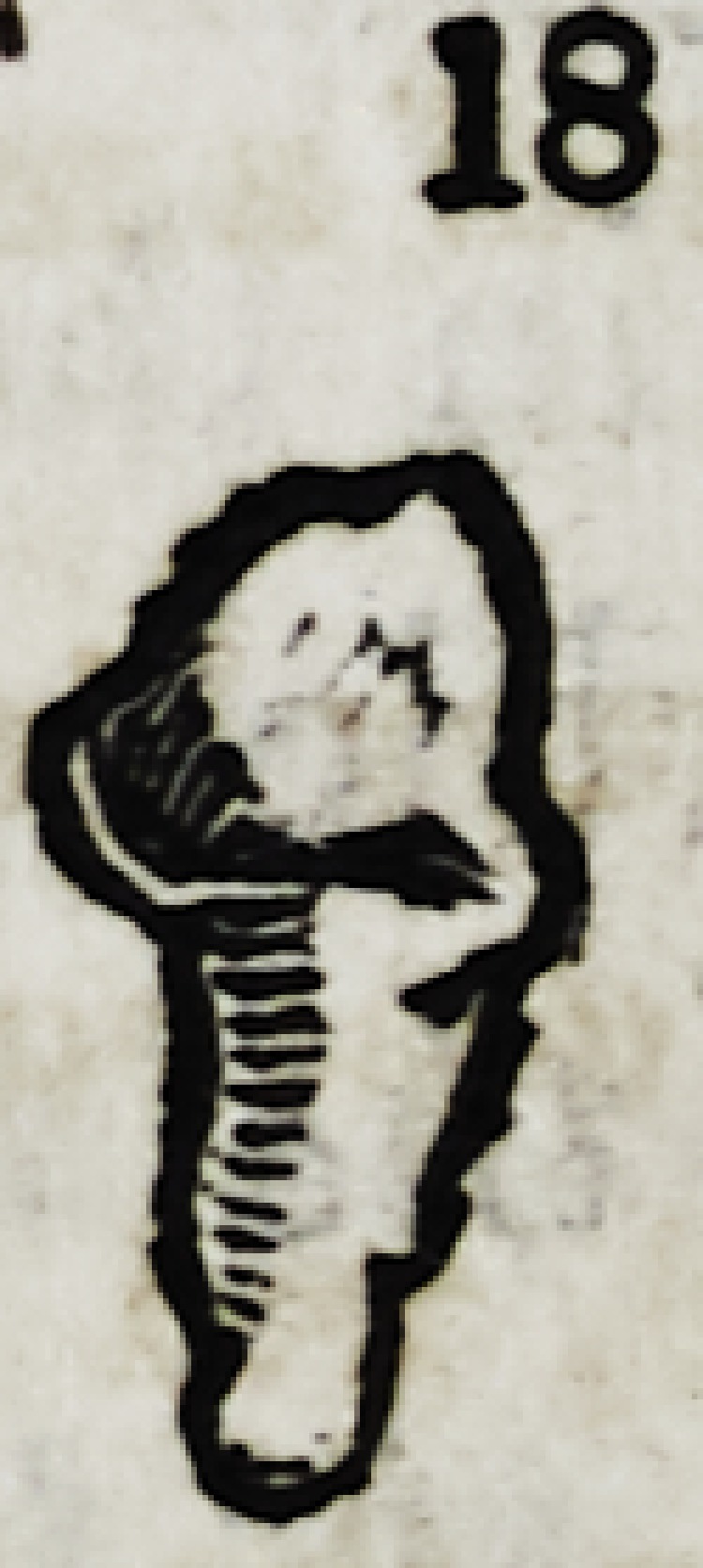# Some Remarks on Supernumerary Teeth

**Published:** 1857-07

**Authors:** J. L. Levison


### AMERICAN JOURNAL OF DENTAL SCIENCE.

Vol. VII. NEW SERIES—JULY, 1857. No. 3.

### ORIGINAL COMMUNICATIONS.

### ARTICLE I.

*Some Remarks on Supernumerary Teeth.* By J. L. Levison, D. D. S., &c., &c., &c.

*To the Editors of Dental Journal:*

Sirs: Although some years since, there was a communication of mine in the Lancet, (December 26th, 1846,) wherein I incidentally alluded to supernumerary teeth, in an article on premature dentition, &c., yet having since had a few cases of the kind, of extreme interest, it may be worthy of the readers of your excellent Journal, to read my additional remarks based on more accurate observations. But as many may not have seen the above paper, I shall repeat a portion of it.

"*Supernumerary Teeth.*—I would also call your attention to another curious fact in the dental system—the formation of supernumerary teeth, which can only be regarded as a connate variety, *similar as in the instances of the occasional development of six fingers and six toes, in some members of a family.*"

Supernumerary teeth are found of various shapes; some conical; some pear shaped; and others resembling teeth of the normal form, somewhat modified in structure. They are usually formed between the central incisors, or between the lateral incisors, (Diagram, 2, 2,) and in other instances in the palatine bones." (Diagram, 12, 13, 15, 16, 17, 18, 20.)*

* This Diagram has been made for the present article.

"There is one great peculiarity, they have usually a greater thickness of enamel than any other teeth in the same mouth. In some instances they are connected with the posterior or the lateral surfaces of the incisor teeth, and in many instances appear as *offshoots* of one of the tooth pulps." (Diagram No. 1.)

"After these statements, a case of a young lady was given, who was very delicate and of a nervous temperament, in which the supernumerary tooth resembled in shape a narrow central incisor, (Diagram 2-2,) the peculiarity of which consisted in a very thick coating of enamel, particularly on its posterior surface, that substance being in thick masses and ribbed."

"There could not be any difficulty in regarding this as a supernumerary tooth, as she had two large central incisors, (one of them being forced in an oblique direction,) with two beautifully formed lateral incisors, on each side of them, besides which, she had the usual number of cuspidati, bicuspids, and molars."—*Lancet, Dec. 26th*, 1846.

Since that time I have met with many extraordinary instances of this freak of nature with other teeth, besides those of the central and the lateral incisors. (Diagram, No. 6.) This is a curious case of a supernumernry *cuspidatus* resembling three distinct palatine supernumerary teeth pressed together. It was found just behind the cuspidatus of the normal charac-

---

### *Explanation of the Diagram.*

FIG. 1. A central incisor tooth, with a distinct *offshoot,* but not detached, otherwise it would have formed a distinct supernumerary tooth.

FIG. 2, 2, 3. Supernumerary lateral incisors.

FIG. 4 and 5. Examples of distorted supernumerary lateral incisors.

FIG. 6. A supernumerary cuspidatus, composed of conical shaped teeth pressed nearly together, yet each in all probability offshoots from a germ of a cuspidatus.

FIG. 7. A supernumerary cuspidatus, rather more conical than the type of the human tooth, as it resembles more that of a canine animal.

FIG. 8. Another cuspidatus of a most remarkable and distorted shape, still retaining something of its type.

FIG. 9 and 10. Supernumerary bicuspids.

FIG. 11. A supernumerary *dens sapientiœ.*

FIG. 12, 13, 14, 15, 16, 17, 18, and 20. Are supernumerary teeth, which were found principally, imbedded in the centre of the palatine bones, ranging from a near proximity to the alveolar arch, to a full inch from the localities of the supposed types.

ter, and the nasal bicuspid tooth, lying rather inward and somewhat obliquely, and produced great irritation. Also 9 and 10 (diagram) are pseudo-bicuspids, taken from two different mouths and which in both instances indicated their abnormal character by being much smaller and shorter than the other and regular formed teeth. They also caused great irritation from not being in any alveolus, but deeply imbedded in the substance of the gums. No. 11 (diagram) is a supernumerary dens sapientiæ, which induced an extensive suppuration in the lower jaw. As in the last mentioned cases, it was not fixed in any alveolus, but was deeply imbedded in the temporal muscles at its union with the coronoid process. This tooth is small, and at its upper surface it resembles three conical palatine teeth (supernumerary ones) compressed together laterally, whilst as a whole it is very short, roundish, with a single fang, and seems to be perfectly solid. It did not induce any marked irritation until the development of the normal *dens sapientiæ* of the same side, which, although the crown was of natural size, had an inward direction into the mouth.

These examples will suffice, as my object is simply to call attention to a few interesting details.

1st. That my former letter in the Lancet is confirmed by subsequent experience; namely, that supernumerary teeth must be regarded as connate varieties. For, in the case of the palatine supernumerary teeth, I have found that often two or three had them in the same family. 2nd. That they must be considered as *lusus naturæ;* for in all the cases I have had, there has not been a single instance in which either parent had any such peculiarity. 3rd. That the incidental remark in the above mentioned letter, that they are portions of the original pulp, is incidentally verified; that each slip being separated from the original germ of a particular tooth is confirmed by the fact, that even with their pseudo shape they have some affinity to their respective types, and that, therefore, that they possess in consequence, an especial interest to the student of nature.

For the primary mucous follicle possessing a determinate type, possesses at the same time a vital formative force, which, when the process is undisturbed, ensures the normal form of the special kind of tooth each follicle represents; and we are warranted in affirming, *that when a portion is accidently separated, if the living organic principle is retained by the offshoot, it continues, even in its imperfect development to assume the form "after its kind," and in obedience to the primary law which governs such formations.* So that we may regard supernumerary teeth as merely *osseous* monstrosities which originate in some casualty, either in the pulps from which they are derived, and that subsequently they are more or less deformed, because the condition of these *offshoots* being more or less imperfect, there is in consequence some modification in the condition of the formative process.

That this is not merely a gratuitous speculation, I may cite the fact that in some few cases, there has been a complete union between the central incisors, the osseous union being induced by a conical shaped supernumerary tooth, which has been itself an offshoot from one of them. So clearly is the law of this formation indicated, that in the latter instance, the connection is clearly traced to the offshoot not being sufficiently detached from its primary source. If it had been, and had retained its vitality, it would have arrived at its comparative maturity as an independent and separate entity. I have recently seen a case where the central incisors were united from behind, that is, on the posterior surface, by means of a conical shaped portion of enamel attached to each.

I am, sir,

Yours, most truly,

J. L. LEVISON.

**19 Dorset Place, Dorset Square, London, *April 2nd,* 1857.**

## Figures and Tables

**2 f1:**
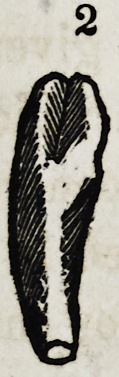


**1-2 f2:**
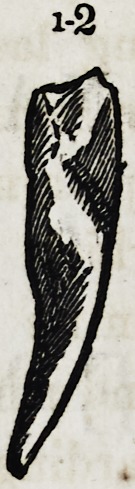


**1 f3:**
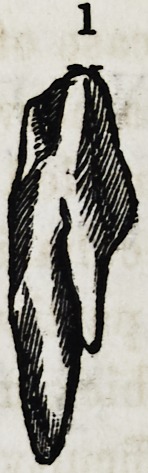


**6 f4:**
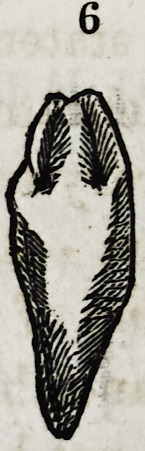


**8 f5:**
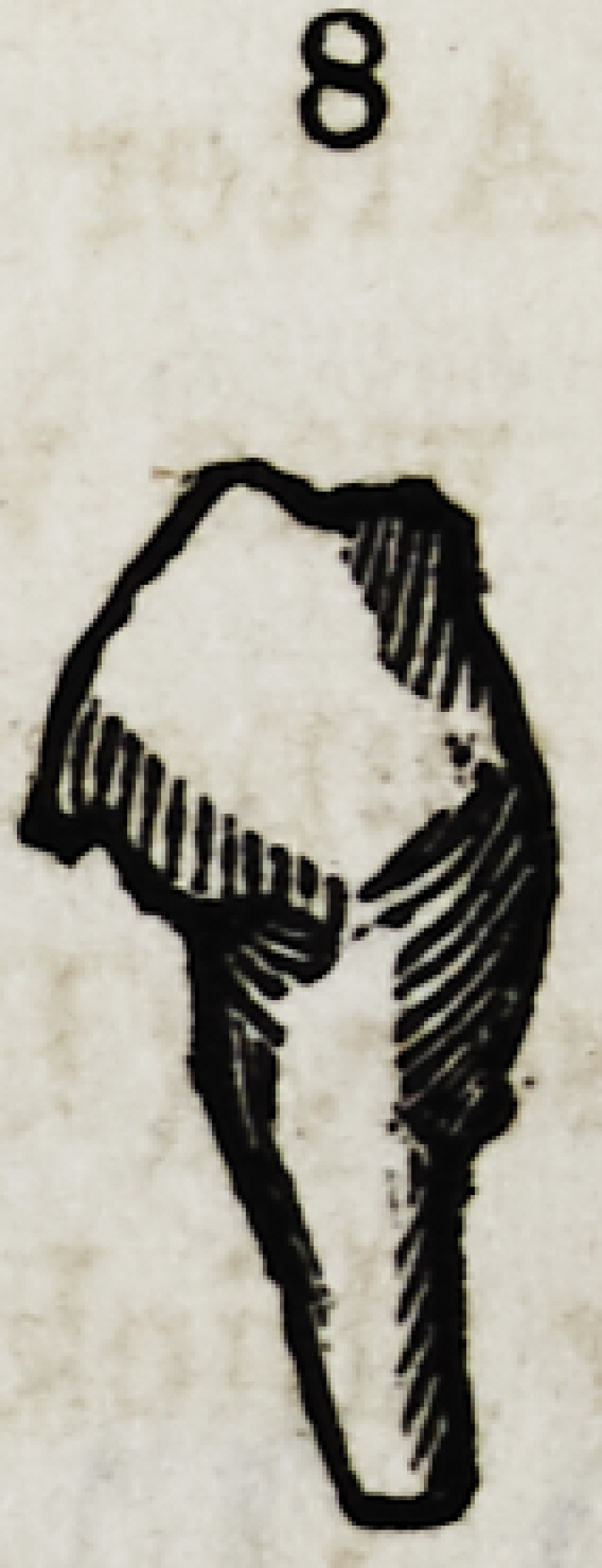


**2-2 f6:**
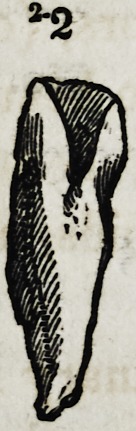


**3 f7:**
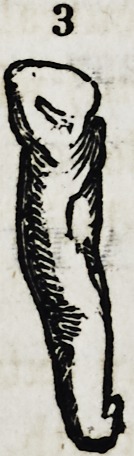


**5 f8:**
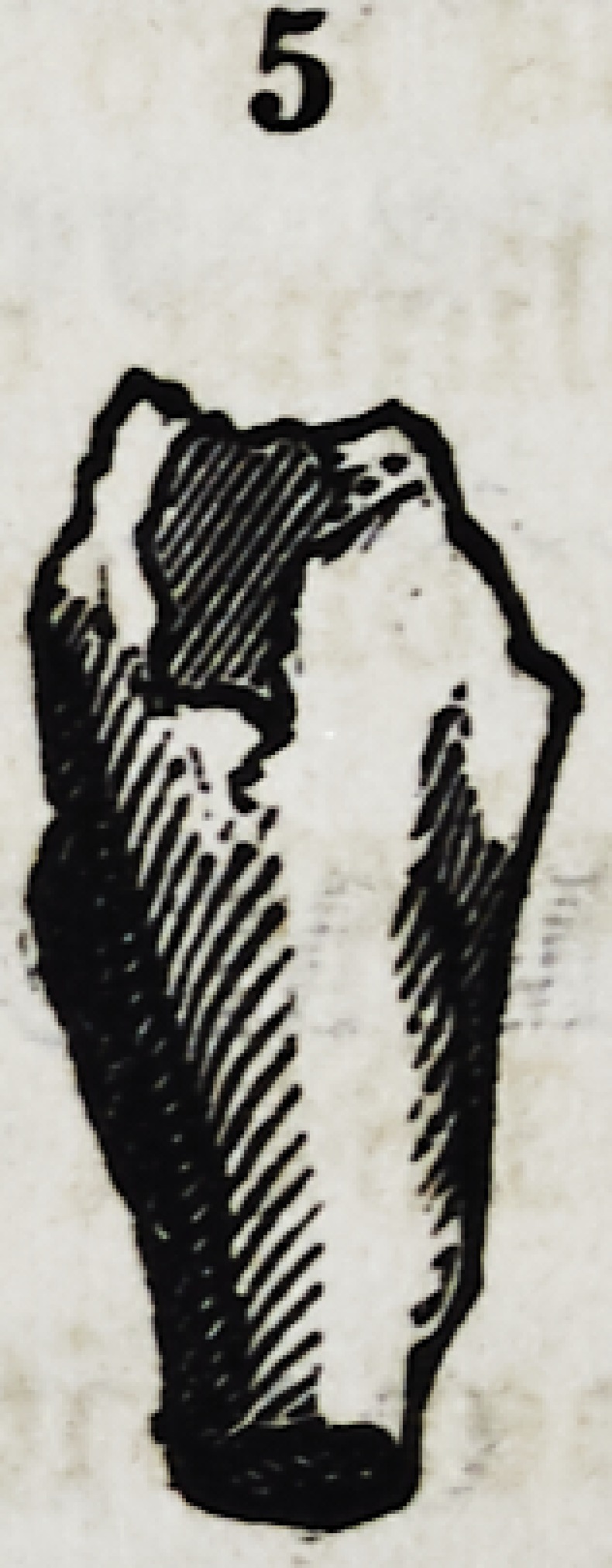


**7 f9:**
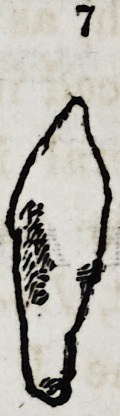


**4 f10:**
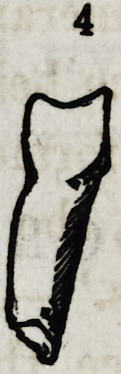


**12 f11:**
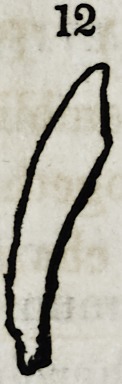


**13 f12:**
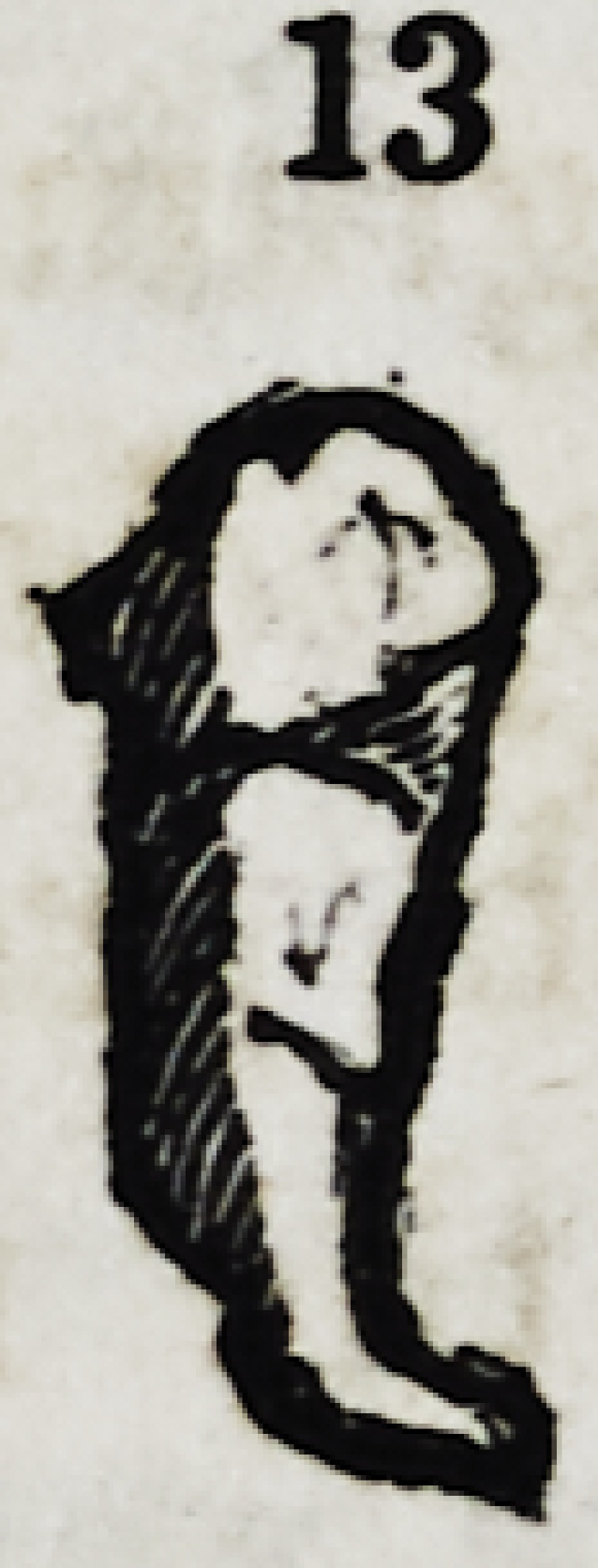


**11 f13:**
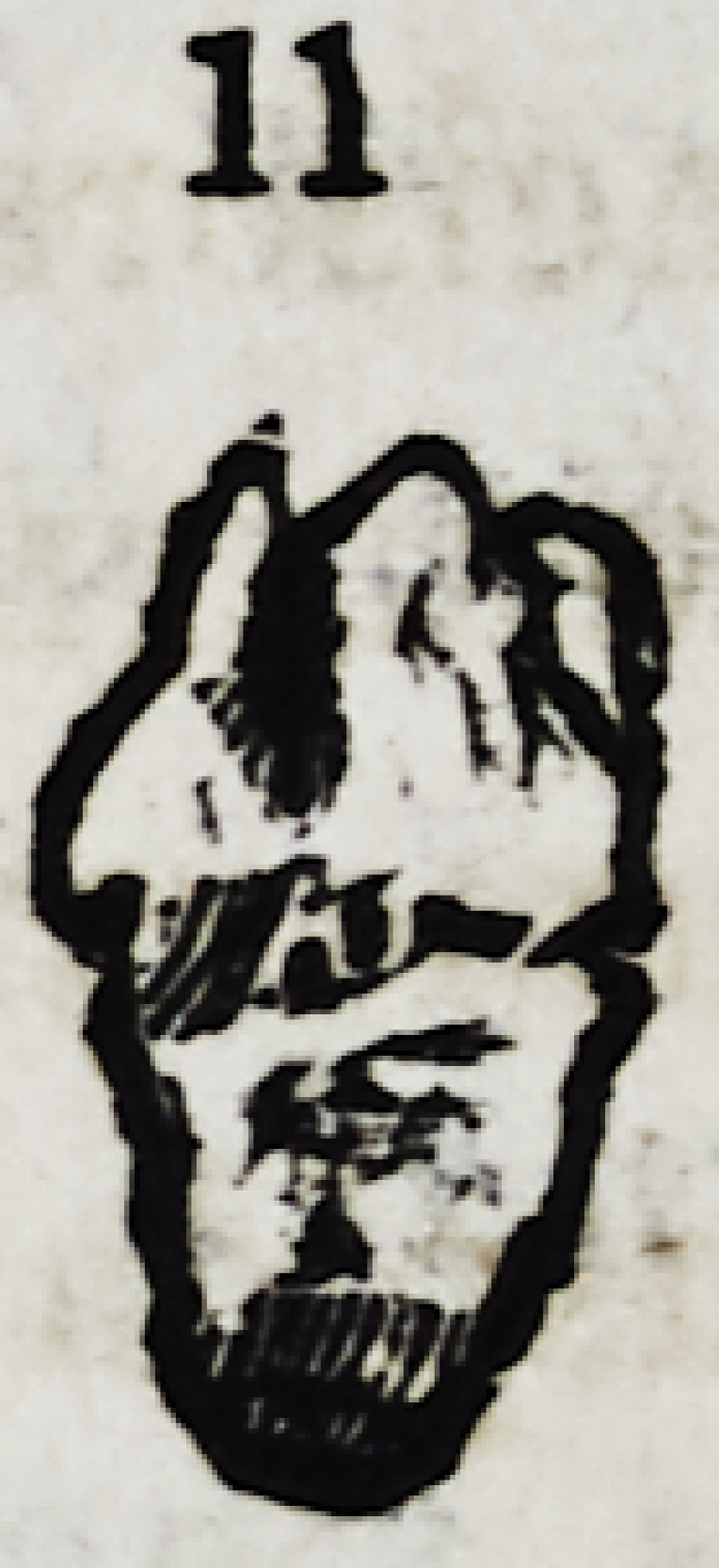


**Figure f14:**
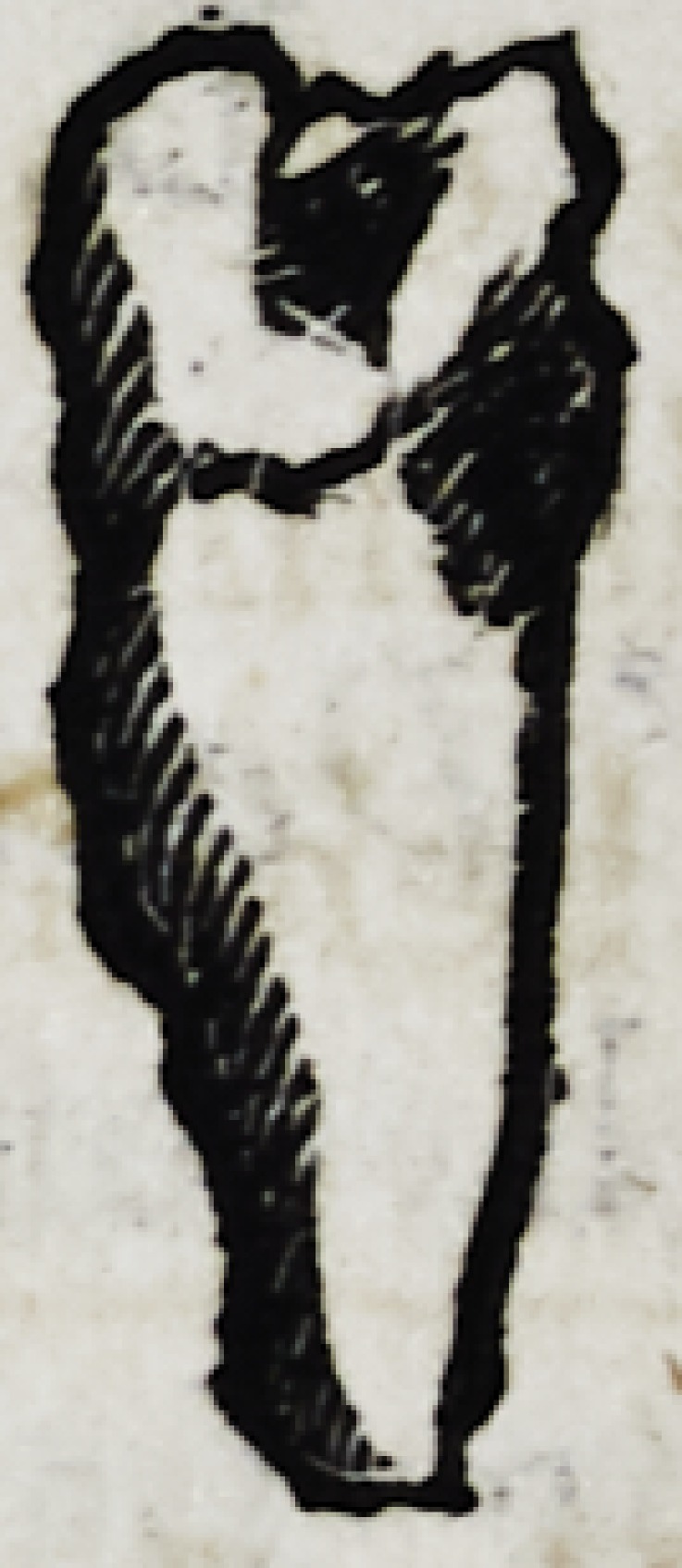


**14 f15:**
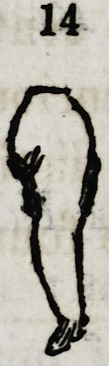


**15 f16:**
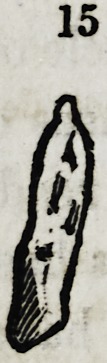


**20 f17:**
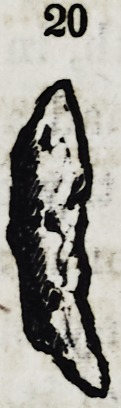


**17 f18:**
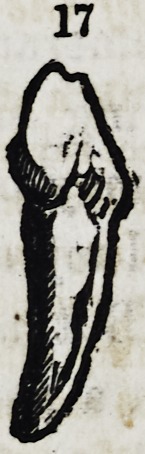


**9 f19:**
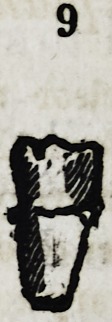


**10 f20:**
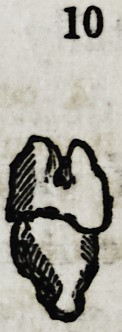


**16 f21:**
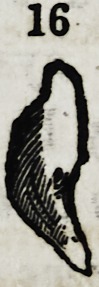


**18 f22:**